# Empathetic leadership and employees’ innovative behavior: examining the roles of career adaptability and uncertainty avoidance

**DOI:** 10.3389/fpsyg.2024.1371936

**Published:** 2024-05-22

**Authors:** Guangya Ma, Weilin Wu, Chenlin Liu, Junhan Ji, Xiaoxiao Gao

**Affiliations:** ^1^School of Foreign Studies, Yiwu Industrial and Commercial College, Yiwu, Zhejiang, China; ^2^School of Economics, Jiaxing University, Jiaxing, Zhejiang, China; ^3^Postdoctoral Workstation, China Rongtong Group Strategy Research Institute, Beijing, China; ^4^School of Economics and Management, Tsinghua University, Beijing, China; ^5^School of Humanities and Tourism, Yiwu Industrial and Commercial College, Yiwu, Zhejiang, China; ^6^School of Business, Shenzhen Institute of Technology, Shenzhen, Guangdong, China

**Keywords:** career adaptability, empathetic leadership, innovative behavior, uncertainty avoidance, career construction theory

## Abstract

**Introduction:**

Career construction theory proposes that adaptivity affects career adapting through career adaptability. However, research on the mechanism of this pathway remains scarce. By applying career construction theory and conservation of resources theory, we hypothesize that career adaptability (concern, control, curiosity, and confidence) mediates the relationship between empathetic leadership (adaptivity) and innovative behavior (career adapting). Moreover, we posit that uncertain avoidance moderates the mediating mechanism.

**Methods:**

Our study used SPSS23 and bootstrap methods (PROCESS) to test the proposed model. The sample comprised 301 employees from different firms in various industries. In this study, empathetic leadership-5, career adaptability-24, uncertainty avoidance-5, and innovative behavior-6 scales were used to measure empathetic leadership, career adaptability, and uncertainty avoidance.

**Results:**

The results revealed that (1) empathetic leadership is positively related to employees’ innovative behavior (2) Concern (H2a), control (H2b), curiosity (H2c), and confidence (H2d) mediate the relations between empathetic leadership and employee’s innovative behavior (3) Uncertainty avoidance moderates the relationship between empathetic leadership and concern (H3a), control (H3b), curiosity (H3c), and confidence (H3d), such that this relationship is stronger when uncertainty avoidance is higher (4) Uncertainty avoidance moderates the indirect relationship between empathetic leadership and employee innovative behavior through concern (H4a), control (H4b), curiosity (H4c), and confidence (H4d), such that this indirect relationship will be stronger when uncertainty avoidance is high than when it is low.

**Conclusion:**

We investigated how empathetic leadership affects innovative behavior. Based on career construction theory and conservation of resources theory, we also tested the hypothesis that concern, control, curiosity, and confidence play mediating roles in linking empathetic leadership (career adaptivity) to innovative behavior (career adapting). In addition, this study found that uncertainty avoidance does not always have negative effects. People with a high uncertainty avoidance tendency may be dependent more on empathetic leadership to improve their career adaptability, which promotes their innovative behavior.

## 1 Introduction

Due to the uncertainty, instability, and ambiguity of the business environment (Wibowo and Paramita, 2022; Yue et al., 2023), the papers about innovation has always underlined the significance of organizations appointing, inspiring, and retaining employees involved in creative activities to maintain the enterprise’s competitive advantage (Scott and Bruce, 1994; Lee et al., 2020). The pandemic may have caused insecurity and anxiety among employees regarding innovation (Hu et al., 2020), which requires understanding and support from leaders.

Empathetic leadership, which requires an emotional support leader, means to an ability to recognize and comprehend the experiences of followers while providing emotional support to make them feel safe (Kock et al., 2019). It requires further research to understand empathy in the workplace (Colbert et al., 2016). At work, an employee can empathize with coworkers, but leaders can offer empathy as well (Kock et al., 2019). This requires leaders to foster connection among their subordinates by understanding human nature and emotional resonance to realize the synergistic development of individuals and organizations. Microsoft CEO Nadella almost lost his chance to join the company as a young man 20 years ago because of empathy. “I want to connect creativity with empathy for others,” he said, “Creativity is exciting, and empathy is at the core of what I do.” At present, empirical research on empathetic leadership is lacking (Bani-Melhem et al., 2021). In order to fill this research blank, we discuss the relationship between empathetic leadership and innovative behavior, which can enrich the research outcome of empathetic leadership.

Although empathetic leadership is known to be conducive to improving employee innovativeness, the mechanism between empathetic leadership and innovativeness among employees has largely been ignored. Kock et al. (2019) believe that job satisfaction, from an affect perspective, is an important mechanism connecting empathetic leadership and innovative behavior. From the perspective of resources, empathetic leadership, as a kind of supportive leadership, may improve innovative behavior by helping individuals increase resources. Therefore, based on career construction theory and conservation of resources theory, this paper introduces career adaptability from the resource perspective to look for the connection mechanism between empathetic leadership and employees’ innovative behavior. Career construction theory suggests that those who are willing (adaptive) and able (adaptability) cope with changing conditions (adaptation), which can advance their careers (Savickas and Porfeli, 2012). Empathetic leadership involves adaptivity and is a predictor of career adaptability. According to conservation of resources theory, employees with sufficient resources will have more time and energy to innovate. Further, career adaptability is a vital supplementary resource for staffs at work, allowing them to cope with work demands (Chan and Mai, 2015). Because people with high career adaptability will obtain sufficient psychological resources, they can adapt to career development by taking proactive actions (Ramos and Lopez, 2018). This study posits that, based on this model, empathetic leadership as adaptive preparation will ultimately affect adaptive behavior by influencing career adaptability resources.

Exploring the conditions under which empathetic leadership increases individual career adaptability can elucidate the role of resource conservation motivation in career construction. Career construction theory reveals the significant impact of the environment (environmental uncertainty) on career adaptability (Rudolph et al., 2019). Environmental factors affect the opportunities and necessary requirement for the development and expression of psychological and societal resources and transactional competencies (Savickas and Porfeli, 2012). Because individuals adopt different attitudes when facing uncertain environments, uncertainty avoidance is the most direct way for individuals to deal with environmental uncertainty. Hiring leaders who are good at adjusting uncertainty in creative work can help overcome the high uncertainty avoidance that employees face when they innovate (Watts et al., 2020). Compared to people with low uncertainty avoidance, people with high uncertainty avoidance face greater pressure (Hofstede, 2001) and are more afraid of taking risks (House et al., 2004). Therefore, individuals with high uncertainty avoidance tend to avoid resource loss through a variety of ways, including relying on the support and help of empathetic leaders to obtain resources.

Our research contributes to understanding innovative behavior in the following areas. First, this study enriches the outcome variables of empathetic leadership (Bani-Melhem et al., 2021). So far, papers on the outcome variable of empathetic leadership is scarce, and our research enriches the literature on this variable. Second, known mediated mechanisms between empathetic leadership and innovative behavior remain scarce. This study considers career adaptability as a mediator, which is beneficial for discussing how empathetic leadership affects employees’ innovative behavior. Karacan-Ozdemir and Ayaz (2022) pointed out that future studies may discuss the mediating role of each adaptability resources through different variables. This study discusses the mediating role of four kinds of adaptive resources in response to the call of Karacan-Ozdemir and Ayaz (2022). Third, although scholars have explored the relationship between empathetic leadership and job innovation (Kock et al., 2019), they have not explored how the relationship between empathetic leadership and job innovation will change under given circumstances. This study considers uncertainty avoidance as a moderator, which is conducive to discussing the relationship between emotional leadership and innovative behavior, complementing previous research on the boundary conditions of the relationship between empathetic leadership and adaptive and innovative behavior. Moreover, most of the previous literature believed that low uncertainty avoidance would enhance the relationship between positive leadership and innovation (Wang, 2020), but this study believes that high uncertainty avoidance would enhance the indirect effect of empathetic leadership on innovation behavior via career adaptability. Such research is helpful to understand the relationship between uncertainty avoidance and leadership. Finally, current research focuses on college students as samples (Zhang et al., 2021), but research on corporate personnel remains inadequate. This study selects enterprise staff as the research object, which is beneficial for enriching the research sample for the career construction model.

## 2 Hypothesis development

### 2.1 Leadership under the theory of career construction and resources conservation

The success of a person’s career is influenced by their social environment (Ng et al., 2005), and individuals can obtain resources to promote job performance from social support. According to the conservation of resources theory, empathetic leadership, as a type of supportive leadership, is conducive to individual career adaptability and positive adaptive results. However, career construction theory does not detail how individuals acquire and use various resources. Therefore, this study draws on the conservation of resources theory (Hobfoll, 2011), as it explains how individuals can use the resources they can access to promote the achievement of goals (Ocampo et al., 2018). According to the conservation of resources theory, individuals achieve valuable goals through conditional resources (supportive working relationships) and personal resources (key skills and personal characteristics). This study explains how empathetic leadership promotes innovative behavior based on career construction theory and conservation of resources theory.

We base our research model on career construction theory. In this model, empathetic leadership, as a type of adaptivity, can influence employees’ career adapting (innovative behavior) through their career adaptability. This construction process is influenced by situational factors. This study uses the conservation of resources theory (Hobfoll, 2011) to explain this construction process. As a type of supportive leadership, empathetic leadership provides employees with resources that benefit their individual work through empathy. Specifically, employees can improve their career adaptability and obtain adaptability resources with the help of leaders. Employees with more resources can obtain additional resources through innovative behavior. Meanwhile, career construction theory points out that environmental factors are important factors that influence personal construction (Savickas and Porfeli, 2012). Uncertainty avoidance is a cultural value that individuals display in the environment. When individuals adopt different attitudes to influence individual innovation behavior in an uncertain environment, engaging leaders skilled in adjusting uncertainty in creative work can help overcome the high uncertainty avoidance faced by employees in innovation (Watts et al., 2020). Because empathic leadership can help employees with high uncertainty avoidance access support and resources, employees with high uncertainty avoidance are likely to rely more on empathetic leadership.

In the following hypothesis development, this study will supplement the explanation of the career construction model through conservation of resources theory, describing how empathetic leadership influences innovative behavior through career adaptability, and the moderating role of uncertainty avoidance.

### 2.2 Empathetic leadership and employees’ innovative behavior

Staff need support, understanding, and empathy in the workplace (Edmondson and Lei, 2014). Article has shown that emotional support can improve employees’ workplace outcomes (Yrie et al., 2003). Empathetic leadership, as exercised by an emotional support leader, means to an ability to recognize and comprehend the experiences of followers while providing emotional support to make others feel safe (Kock et al., 2019). Empathetic leadership proclaims if leaders can manage better when they know the emotional characteristics of their subordinates, express this understanding, and support them in dealing with these emotions. This makes followers will better about their work conditions, thereby improving their performance (Kock et al., 2019). Thus, empathetic leaders in the workplace foster a positive state among followers (Wibowo and Paramita, 2022) and themselves (Boyatzis et al., 2006).

Conservation of resources theory suggests that people have the motivation to strive toward obtaining and preserving resources (Hobfoll, 1998), and they must invest resources to prevent the loss of resources (Hobfoll, 2011). First, in providing employees with instructional support resources (Bani-Melhem et al., 2021), empathetic leaders attach great importance to the personal needs of employees (Kock et al., 2019) and even provide instruction resources when employees encounter problems. After employees receive the resources from the leaders, to obtain more resources and prevent resource loss, employees give full play to their resource advantages and improve their innovativeness through the resources provided by the leaders (Iqbal et al., 2020). In addition, people with more resources are more likely to obtain new resources (Hobfoll, 2011). By encouraging employees to express their ideas, leaders make followers more confident and reduce their fear of innovative behavior (Lee et al., 2020), thereby promoting innovative behaviors.

Second, empathetic leaders provide employees with emotional support resources (Bani-Melhem et al., 2021), and they integrate emotions into the communication process to express concern for employees (Mayfield and Mayfield, 2017a). This results in employees have a good sense about their work situation (Kock et al., 2019) and believing their leader as warm. This increased trust and positive affect among employees results in them developing new ideas and being more innovative to obtain more emotional resources from leaders (Hobfoll, 2011). Accordingly, we propose the hypothesis:

*H1*: Empathetic leadership is positively related to employees’ innovative behavior.

### 2.3 Mediating role of career adaptability

Career construction theory proposes a career construction model that explains the career-building process over a person’s life span through the relationship among adaptivity, adaptability, adapting, and adaptation dimensions. Career adaptivity is defined as a psychological feature, that is, be having the flexibility and willingness to adapt to facilitate proactive attempts to address responsibilities related to professional development as well as job transition and employment issues (Savickas and Porfeli, 2012). The psychological and social framework of career adaptability describes the tools people need to deal with expected and existing demands, changes, and traumas in their professional roles (Savickas, 1997). Career adapting (adapting responses) refers to behaviors involving changing career conditions and making career choices (Sverko and Babarovic, 2019). Career adaptation (adaptation results) is the condition realized through the career construction process, so it refers to career outcomes (Sverko and Babarovic, 2019). With respect to the use of career construction theory, academics categorize under career adaptability cognitive talents, fundamental personality traits, future orientation, dispositional positivity, openness to experience, conscientiousness, and career-related support (Rudolph et al., 2017a; Tokar et al., 2020; Zhang et al., 2021). Adaptation preparation is expected to promote the development of career adaptability resources (Sverko and Babarovic, 2019). In turn, career adaptability resources can promote positive career adapting (Hirschi et al., 2015; Peng et al., 2021) and career adaptation (Rudolph et al., 2017b). Career construction theory points out that career adaptability, as a psychological resource, can help individuals cope with and solve unfamiliar and complex problems encountered in their careers. It consists of four main resources: concern (preparation for future careers), control (people’s perceived ability to manage their careers), curiosity (exploration of career opportunities), and confidence (determination to overcome career difficulties) (Savickas, 2002). Based on the career construction model and theory, this study constructs a mediation model for empathetic leadership (career adaptivity), career adaptability, and innovative behavior (career adapting).

In accordance with career construction theory and conservation of resources theory, leaders are regarded by employees through the lens of adaptivity in their search for career adaptability resources (Al-Ghazali, 2020; Lan and Chen, 2020), and employees can complete work tasks (career adapting) by utilizing adaptability resources. First, empathetic leaders support their subordinates by expressing empathy for them (Cornelis et al., 2013), and this active mood relationships makes employees feel that leaders respect them and consider their needs (Kock et al., 2019). This helps subordinates explore future needs and prepare for future career tasks (Al-Ghazali, 2020). Second, empathetic leaders enhance their ability to make career decisions, improve work skills and their sense of responsibility and control over work. Third, when empathetic leaders support subordinates (Kock et al., 2019), the latter use the tools supplied by leaders to adjust to changes in their careers and resolve issues at work (Savickas, 2005). Moreover, to obtain more support from leaders, employees will improve their ability to actively explore the environment and opportunities (Hobfoll, 2011). Fourth, the emotional support leaders offer increases trust between them and their subordinates (Mayfield and Mayfield, 2017c), creates a good working environment for employees (Kock et al., 2019), and boosts the subordinates’ confidence. These factors improve employees’ adaptability.

On the other hand, career adaptability recognized as a type of psychological resource (Savickas and Porfeli, 2012) boosts predict employees’ work behavior (career adapting) (Lan and Chen, 2020). Concern, control, curiosity, and confidence are the four critical resources via which career adaptability fosters innovative behavior. First, employees focus on the future, plan and prepare for their future careers, and develop new ideas and methods for innovation. Second, they do not mind taking chances when innovation fails since they feel in charge of the future. Third, they are curious about the future and dare to take risks and innovate. Fourth, they are full of confidence in their ability to solve innovation-related problems and get over career development challenges (Savickas, 2013). Therefore, career adaptability can help employees in channeling their self-regulation ability toward solving the unclear and complex problems they will encounter in their career (Savickas and Porfeli, 2012), and employees have more resources for implementing innovativeness (Hobfoll, 2011).

Thus, this study considers that empathetic leadership creates a good working environment for employees by providing them with emotional support and helping them obtain more adaptability resources. To obtain more support from leaders, employees will incorporate adaptability resources into their work, thus increasing innovative behavior. The following hypotheses are formulated:

*H2*: Concern (H2a), Control (H2b), Curiosity (H2c), and Confidence (H2d) mediate the relations between empathetic leadership and employee’s innovative behavior.

### 2.4 Moderating role of uncertainty avoidance

Uncertainty avoidance is of the opinion that the degree to which a society perceived menace by non-determinacy and equivocal circumstances, and attempts to prevent these circumstances by providing greater professional steadiness, setting up more official provision, not enduring distinctive thoughts and actions, having faith in complete truth, and acquiring professional knowledge (Hofstede, 1980c). To avoid uncertain situations, people will try to establish official rules and reject deviant thoughts and behaviors (Hofstede, 1980b). Although Hofstede (1980a) later defined uncertainty avoidance based on social level, many scholars have stated that opinion about cultural values (uncertainty avoidance) can occur at the individual level (Afsar and Masood, 2018; Cai et al., 2020) and have a great impact on personal attitudes and behaviors (Afsar and Masood, 2018). This study suggests that different standards of personal uncertainty avoidance influence the research of empathetic leadership and career adaptability.

Conservation of resources theory suggests that individuals have a motivation to protect resources and prevent resource loss, that is, they will participate in behaviors to avoid resource loss (Halbesleben et al., 2014). Compare with people with low uncertainty avoidance, people with high uncertainty avoidance tend to experience more pressure in the presence of ambiguity and uncertainty (Hofstede, 2001). As a result, these people might be more driven to gather and wisely employ different work resources (Jang et al., 2018) in order to prevent resource loss and increase resource availability. High uncertainty avoidance employees are more inclined to look on the organization’s leaders for guidance (Cai et al., 2020). Accordingly, due to the motivation to obtain resources and prevent resource loss (Hobfoll, 2011), employees require empathetic support to obtain adaptability resources. Empathetic leadership is an effective channel and source for employees to obtain emotional support, trust, and feelings of safety (Kock et al., 2019), which can lead to higher career adaptability. Employees rely more on the support of empathetic leaders to help them constantly seek new opportunities to develop new career goals (concern); Enabling individuals to take decisive action to overcome unexpected difficulties (control); Exploring future career roles (curiosity); Enables individuals to set challenging goals and work toward them consistently (confidence) (Savickas, 2013; Yang et al., 2015). In contrast, employees with low uncertainty avoidance are not restricted by rules and dare to take risks and explore. They have strong internal motivation and are willing to obtain resources for themselves through risk-taking and initiative. Therefore, these employees are less sensitive to the support provided by empathetic leadership and are less likely to obtain career adaptability from leaders. In summary, we assume the following:

*H3*: Uncertainty avoidance moderates the relationship between empathetic leadership and concern (H3a), control (H3b), curiosity (H3c), and confidence (H3d), such that this relationship is stronger when uncertainty avoidance is higher.

### 2.5 Mediation model with moderation

Our paper concludes that uncertainty avoidance moderates the indirect relationship between empathetic leadership and employee innovative behavior through career adaptability by taking into account the analytical structure of moderated mediation (Preacher et al., 2007), combining H2 and H3a. Loss aversion and the resulting motivation for resource conservation are two key processes in conservation of resources theory (Halbesleben et al., 2014). According to career construction theory and conservation of resources theory, employees with uncertainty avoidance emphasize predictability and clear instructions (Triandis, 1990) and feel that the threat of uncertainty and ambiguity is high (Hofstede, 2001). They are afraid of failure and resource loss and focus on security. To reduce this threat, employees will take action. Because of the motivation to protect resources, employees with high uncertainty avoidance are more receptive to leadership support (Cai et al., 2020). They focus on obtaining adaptability resources through the support of sympathetic leaders. Employees can seriously consider and prepare for future career possibilities (concern), make decisions and take serious action (control), explore future career scenarios (curiosity) and have a positive perception of their ability to solve problems (confidence) (Savickas and Porfeli, 2012). People with this greater career adaptability are more willing to participate in innovative behaviors that try to alter work features, which can lead to the acquisition of additional resources (Federici et al., 2021). Employees can also direct their attention, motivation, and behavioral engagement through professional resilience, updating their professional self-concept to meet the adaptive requirements of career development (Ramos and Lopez, 2018), thereby increasing their innovative behavior. Employees with low uncertainty avoidance do not need specific resources from leaders to reduce uncertainty in their work but are less sensitive to the support of sympathetic leaders. Therefore, they cannot improve their professional adaptability and increase their innovative behavior.

*H4*: Uncertainty avoidance moderates the indirect relationship between empathetic leadership and employee innovative behavior through concern (H4a), control (H4b), curiosity (H4c), and confidence (H4d), such that this indirect relationship will be stronger when uncertainty avoidance is high than when it is low.

## 3 Research methods

### 3.1 Sample and procedure

Our paper considered Chinese employees and recruited participants from different firms in various industries (financial, manufacturing, IT, services, and real estate industries) in Guangdong, Zhejiang, and Henan, in China, aiming to improve innovative activities in the workplace. Specifically, these companies and their employees face a constant need for product, technology, and service innovation (Odoardi et al., 2015). For example, employees in the finance, real estate and service industries need to provide customized services to different customers according to their needs. Employees in manufacturing and technology industries need to design and implement engineering or software products tailored to customer needs. In all industries, managers are also required to support the innovative behavior of their subordinates. Therefore, this study selected data from financial, manufacturing, IT, services, and real estate industries to investigate leadership and innovative behavior.

To avoid the potential common method biases, we utilized a supervisor–employee pairing format and collected data in three stages (with a one-month interval between each stage). After the researchers and HR managers contacted the direct supervisor, the supervisor and employees were informed of the purpose of this study, the questionnaire filling process, and confidentiality agreements. Afterward, on-site questionnaires were distributed. Once these were completed, they were handed over to the researchers. The 500 questionnaires distributed at time 1 were filled by employees and collected information on empathetic leadership and uncertainty avoidance. Career adaptability questionnaires were administered at time 2 to employees who received 462 questionnaires in time 1. The 423 employees were named after the questionnaire was collected at time 2, and the innovative behavior data of these 423 employees evaluated by their supervisors were collected in time 3. Three-stage data matching was conducted according to the last four digits of the employee phone number reserved by the employee and the supervisor. Finally, 301 valid questionnaires were obtained after the invalid questionnaires were eliminated, with an effective recovery rate of 60.2%.

Among the 301 valid paired samples, the IT industry accounted for the largest proportion, accounting for 44.5% (134 respondents); 59.5% (179 respondents) were males. In terms of education level, 130 respondents had a high school degree or below, and 171 respondents had a bachelor’s degree or above. In terms of marital status, the majority (277 respondents) were married. In terms of work experience, 104 respondents worked less than 5 years, and 142 respondents worked for 6–10 years.

### 3.2 Measures

In this study, the mature scale was selected to measure the variables. To ensure accurate and precise translation, the scale followed the back-translation procedure recommended by Brislin (1980), and the translated Chinese version was proofread. Except for the control variables, the variables considered in this study all used the Likert 5-point scale, with 1–5 ranging from “strongly disagree” to “strongly agree.”

#### 3.2.1 Empathetic leadership

The scale of five items prepared by Kock et al. (2019) was adopted, which passed the empathetic section of the motivating language scale to measure empathetic leadership (Mayfield and Mayfield, 2017b). Representative topics include “My supervisor shows me encouragement for my work efforts.” Cronbach’s alpha for empathetic leadership was 0.91.

#### 3.2.2 Career adaptability

Career adaptability was assessed using Savickas and Porfeli’s (2012) 24-item scale comprising four dimensions (concern, control, curiosity, and confidence). An example item was “Looking for opportunities to grow as a person.” Cronbach’s alpha for career adaptability was 0.97.

#### 3.2.3 Uncertainty avoidance

Uncertainty avoidance was measured using a five-item scale by Youngdahl et al. (2003). An example was “It is important to closely follow instructions and procedures.” Cronbach’s alpha for uncertainty avoidance was 0.92.

#### 3.2.4 Innovative behavior

We used the 6-item scale developed by Scott and Bruce (1994). A sample item was “Investigates and secures funds needed to important new ideas.” Cronbach’s alpha for innovative behavior was 0.87.

#### 3.2.5 Control variables

We controlled for gender (0 = male, 1 = female), marital status (0 = single or divorced, 1 = married), and the employees’ years of work experience (tenure), as research has demonstrated that these three variables are related to employees’ innovative behavior (Wang et al., 2015; Bani-Melhem et al., 2018; Newman et al., 2018).

## 4 Research results

### 4.1 Confirmatory factor analysis and discriminant validity

As the hypothesis of this study involves four dimensions of career adaptability, the confirmatory factor analysis (CFA) includes seven factors. The results obtained through Mplus 8.3 are listed in Table 1. The seven-factor model has good model fit (RMSEA = 0.038, SRMR = 0.037, CFI = 0.963, TLI = 0.960). Moreover, the model fit index is better than that of other competitive models. Noticeably, the variables measured in this study have good discriminant validity. This study also adopted the unmeasured latent market construct (ULMC) approach suggested by Bagozzi and Yi (1988) to test common method variance. As shown in Table 1, the model fit indexes between the two models did not show a large difference (∆CFI = 0.005, ∆TLI = 0.006, ∆RMSEA = 0.002, ∆SRMR = 0.04), no more than the threshold of 0.050 (Bagozzi and Yi, 1988).

**Table 1:** Results of confirmatory factor analysis (CFA).

Models	*χ^2^*	*df*	Δ*x^2^*	RMSEA	SRMR	CFI	TLI
CMV+ A + B1 + B2 + B3 + B4 + C + D	1070.615	718		0.040	0.076	0.958	0.954
A + B1 + B2 + B3 + B4 + C + D	1026.874	719		0.038	0.037	0.963	0.960
A + C, B1, B2, B3, B4, D	1627.852	725	600.978***	0.064	0.060	0.892	0.883
A + D, B1, B2, B3, B4, C	1580.736	725	553.862***	0.063	0.082	0.897	0.889
C + D, A, B1, B2, B3, B4	1848.491	725	821.617***	0.072	0.139	0.865	0.855
A+ B1 + B2 + B3 + B4, C, D	2205.102	737	1178.228***	0.081	0.081	0.824	0.813
B1 + B2 + B3 + B4 + C, A, D	2363.756	737	1336.882***	0.086	0.087	0.805	0.793
B1 + B2 + B3 + B4 + D, A, C	1691.767	737	664.893***	0.066	0.052	0.885	0.879
A, B1, B2, B3, B4, C, D	3347.499	740	2320.625***	0.108	0.103	0.687	0.670

*N* = 301; ***p < 0.001; A, empathetic leadership; B1, concern; B2, control; B3, curiosity; B4, confidence; C, uncertainty avoidance; D, innovative behavior; RMSEA, root mean square error of approximation; SRMR, standardized root-mean-square residual; CFI, comparative fit index; TLI, Tucker–Lewis index.

We tested convergent validities (CR and AVE). The results in Table 2 reveal that the AVE of all constructs exceeded the benchmark of 0.50, and CR exceeded 0.70 (Hair et al., 2010). These results indicate that the study has good convergent validities.

**Table 2:** Results of AVE and CR.

Constructs	*α*	AVE	CR
Empathetic leadership	0.908	0.665	0.908
Career adaptability -concern	0.902	0.611	0.904
Career adaptability -control	0.888	0.575	0.890
Career adaptability -curiosity	0.898	0.602	0.901
Career adaptability -confidence	0.907	0.625	0.909
Uncertainty avoidance	0.918	0.690	0.918
Innovative behavior	0.872	0.531	0.872

α, Cronbach’s alpha; CR, composite reliability; AVE, average variance extracted; MSV, maximum shared variance; ASV, average shared variance.

### 4.2 Descriptive statistics and correlations

The descriptive statistics and correlations of variables are listed in Table 3. Empathetic leadership was positively correlated with career adaptability (*r* = 0.44, *p* < 0.01), concern (*r* = 0.39, *p* < 0.01), control (*r* = 0.38, *p* < 0.01), curiosity (*r* = 0.43, *p* < 0.01), confidence (*r* = 0.42, *p* < 0.01), and innovative behavior (*r* = 0.48, *p* < 0.01). Career adaptability (*r* = 0.69, *p* < 0.01), concern (*r* = 0.62, *p* < 0.01), control (*r* = 0.65, *p* < 0.01), curiosity (*r* = 0.65, *p* < 0.01), and confidence (*r* = 0.62, *p* < 0.01) were positively correlated with innovative behavior. These results provide preliminary support for hypothesis testing.

**Table 3:** Descriptive statistics and correlations for variables (*N* = 301).

Variables	Mean	SD	1	2	3	4	5	6	7	8	9	10	11
1. Gender	0.59	0.49											
2. marital	0.82	0.39	0.01										
3. Tenure	9.27	5.54	0.02	0.32**									
4. EL	3.31	0.98	−0.01	0.04	0.08	(0.91)							
5. CA	3.05	0.86	0.03	−0.07	−0.04	0.44**	(0.97)						
6. Concern	3.04	0.93	−0.01	−0.07	−0.04	0.39**	0.90**	(0.90)					
7. Control	3.05	0.90	−0.01	−0.03	−0.04	0.38**	0.93**	0.81**	(0.89)				
8. Curiosity	3.06	0.93	0.06	−0.07	−0.02	0.43**	0.94**	0.77**	0.85**	(0.90)			
9. Confidence	3.06	0.99	0.08	−0.07	−0.05	0.42**	0.90**	0.71**	0.75**	0.82**	(0.91)		
10. UA	2.91	1.25	−0.02	−0.04	−0.02	0.52**	0.34**	0.27**	0.31**	0.32**	0.34**	(0.92)	
11. IB	3.39	0.77	0.03	−0.07	0.01	0.48**	0.69**	0.62**	0.65**	0.65**	0.62**	0.29**	(0.87)

*N* = 301; *p < 0.05; **p < 0.01; ***p < 0.001; EL, empathetic leadership; CA, career adaptability; UA, uncertainty avoidance; IB, innovative behavior.

### 4.3 Hypothesis testing results

We tested hypothesis 1, 2, and 3 via multiple regression analyses. Tables 4, 5 present the results obtained from SPSS23 and bootstrap methods (PROCESS). We used 5,000 bootstrap samples to estimate indirect effects to create bias-corrected confidence intervals (CIs) (Hayes, 2017). As listed in M17 of Table 5, empathetic leadership was positively and significantly related to employee’s innovative behavior (*r* = 0.38, *p* < 0.001), which supports hypothesis 1.

**Table 4:** Results of regression analysis of career adaptability (concern, control, curiosity, confidence).

	CA	CA	CA	Concern	Concern	Concern	Control	Control	Control	Curiosity	Curiosity	Curiosity	Confidence	Confidence	Confidence
	M1	M2	M3	M4	M5	M6	M7	M8	M9	M10	M11	M12	M13	M14	M15
Gender	0.07	0.07	0.07	−0.02	−0.01	−0.02	−0.01	−0.00	−0.01	0.12	0.13	0.13	0.15	0.16	0.16
Marital	−0.13	−0.15	−0.17	−0.17	−0.19	−0.21	−0.05	−0.07	−0.09	−0.18	−0.19	−0.21	−0.14	−0.16	−0.18
Tenure	−0.00	−0.01	−0.01	−0.00	−0.01	−0.01	−0.01	−0.01	−0.01	0.01	−0.01	−0.00	−0.01	−0.01	−0.01
EL		0.40***	0.16		0.38***	0.16		0.36***	0.11		0.42***	0.19*		0.43***	0.19*
UA			−0.17			−0.22			−0.17			−0.17			−0.13
EL* UA			0.11*			0.11*			0.11*			0.11*			0.10*
*R^2^*	0.01	0.21	0.24	0.01	0.16	0.18	0.00	0.15	0.18	0.01	0.20	0.23	0.01	0.19	0.22

*N* = 301; *p < 0.05; **p < 0.01; ***p < 0.001; EL, empathetic leadership; CA, career adaptability; UA, uncertainty avoidance.

**Table 5:** Results of regression analysis of innovative behavior (IB).

	IB											
	M16	M17	M18	M19	M20	M21	M22	M23	M24	M25	M26	M27
Gender	0.05	0.06	0.02	0.06	0.06	−0.01	−0.02	0.02	0.06	0.06	0.00	−0.00
Marital	−0.16	−0.18	−0.08	−0.08	−0.14*	−0.07	−0.10	−0.10	−0.11	−0.15	−0.10	−0.12
Tenure	0.01	0.00	0.01	0.01	0.01	0.01	0.01	0.01	0.00	0.01	0.00	0.01
EL		0.38***						0.17***	0.22***	0.22***	0.19***	0.21***
CA			0.62***					0.54***				
Concern				0.51***					0.42***			
Control					0.56***					0.47***		
Curiosity						0.54***					0.45***	
Confidence							0.48***					0.39***
*R^2^*	0.01	0.24	0.48	0.39	0.43	0.43	0.39	0.52	0.45	0.49	0.48	0.44

*N* = 301; *p < 0.05; **p < 0.01; ***p < 0.001; IB, innovative behavior; EL, empathetic leadership; CA, career adaptability; UA, uncertainty avoidance.

Hypothesis 2 proposes concern (H2a), control (H2b), curiosity (H2c), and confidence (H2d) as mediators of the relationship between empathetic leadership and employees’ innovative behavior. The research results are listed in Table 6, which reveals that career adaptability mediates the relationship between empathetic leadership and innovative behavior (*B* = 0.21, SE = 0.03, CI = [0.16, 0.27]). Meanwhile, in Table 5, M23 regressed both empathetic leadership (*r* = 0.17, *p* < 0.01) and career adaptability (*r* = 0.54, *p* < 0.001) for innovative behavior, thus weakening the positive relationship between empathetic leadership and innovative behavior. This confirms that career adaptability partially mediated the relationship between empathetic leadership and innovative behavior. Similarly, concern (Table 6, *B* = 0.16, SE = 0.03, CI = [0.11, 0.21]), control (Table 6, *B* = 0.17, SE = 0.03, CI = [0.12, 0.22]), curiosity (Table 6, *B* = 0.19, SE = 0.03, CI = [0.14, 0.24]), and confidence (Table 6, *B* = 0.17, SE = 0.02, CI = [0.13, 0.22]) play a mediating role between empathetic leadership and innovative behavior. According to Table 5 (M24, M25, M26, and M27), concern (*r* = 0.42, *p* < 0.001), control (*r* = 0.47, *p* < 0.001), curiosity (*r* = 0.45, *p* < 0.001) and confidence (*r* = 0.39, *p* < 0.001) partially mediated the relationship between empathetic leadership and innovative behavior, thereby supporting hypothesis 2a, 2b, 2c, and 2d.

**Table 6:** Mediation effects.

Model Pathways	Indirect effect	SE	95%CI
EL → CA → IB	0.21	0.03	[0.16, 0.27]
EL → Concern→IB	0.16	0.03	[0.11, 0.21]
EL → Control→IB	0.17	0.03	[0.12, 0.22]
EL → Curiosity→IB	0.19	0.03	[0.14, 0.24]
EL → Confidence→IB	0.17	0.02	[0.13, 0.22]

*N* = 301; EL, empathetic leadership; CA, career adaptability; IB, innovative behavior.

**Figure 1:**
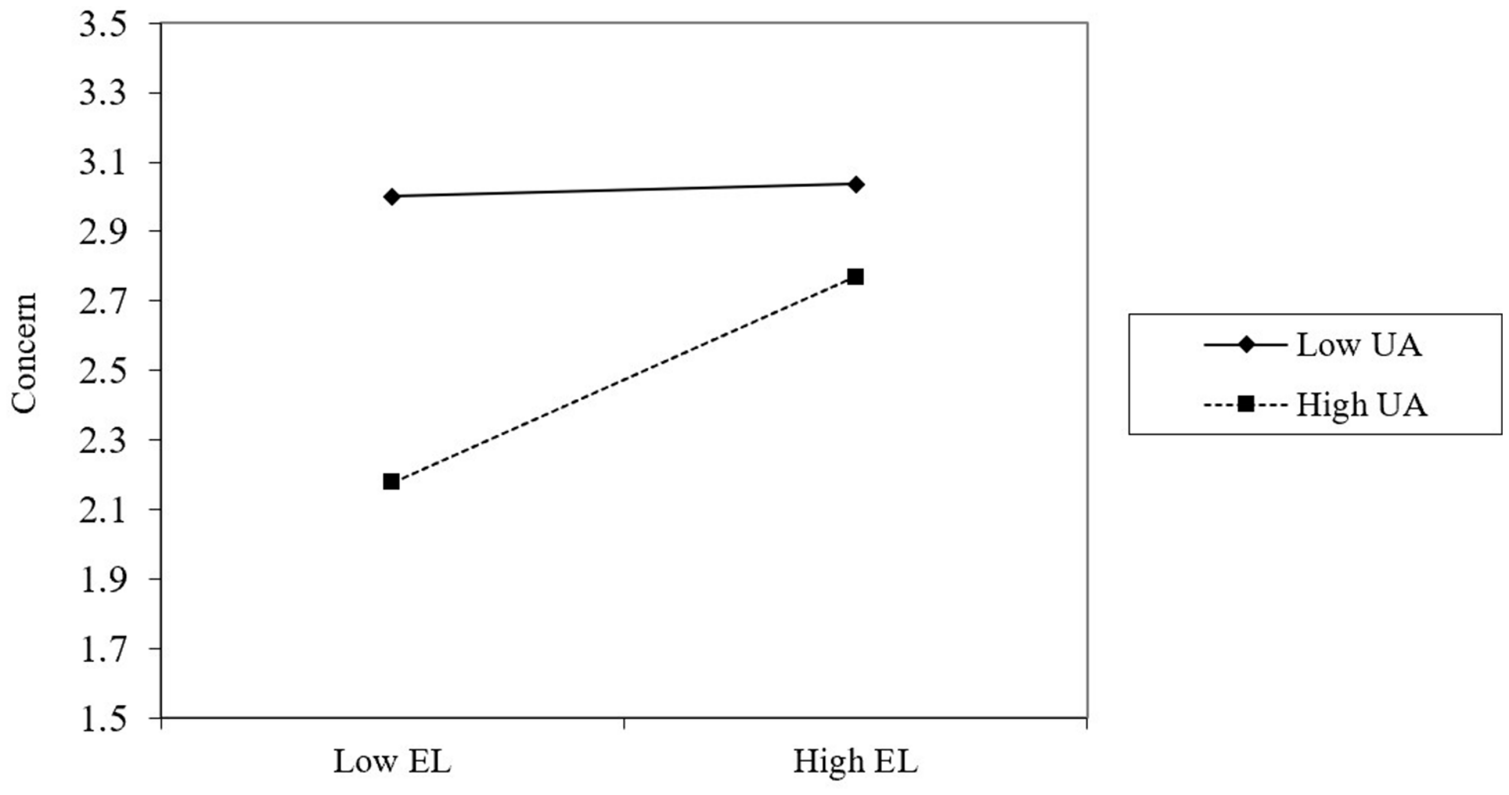
Uncertainty avoidance moderates the relationship between empathetic leadership and concern.

**Figure 2:**
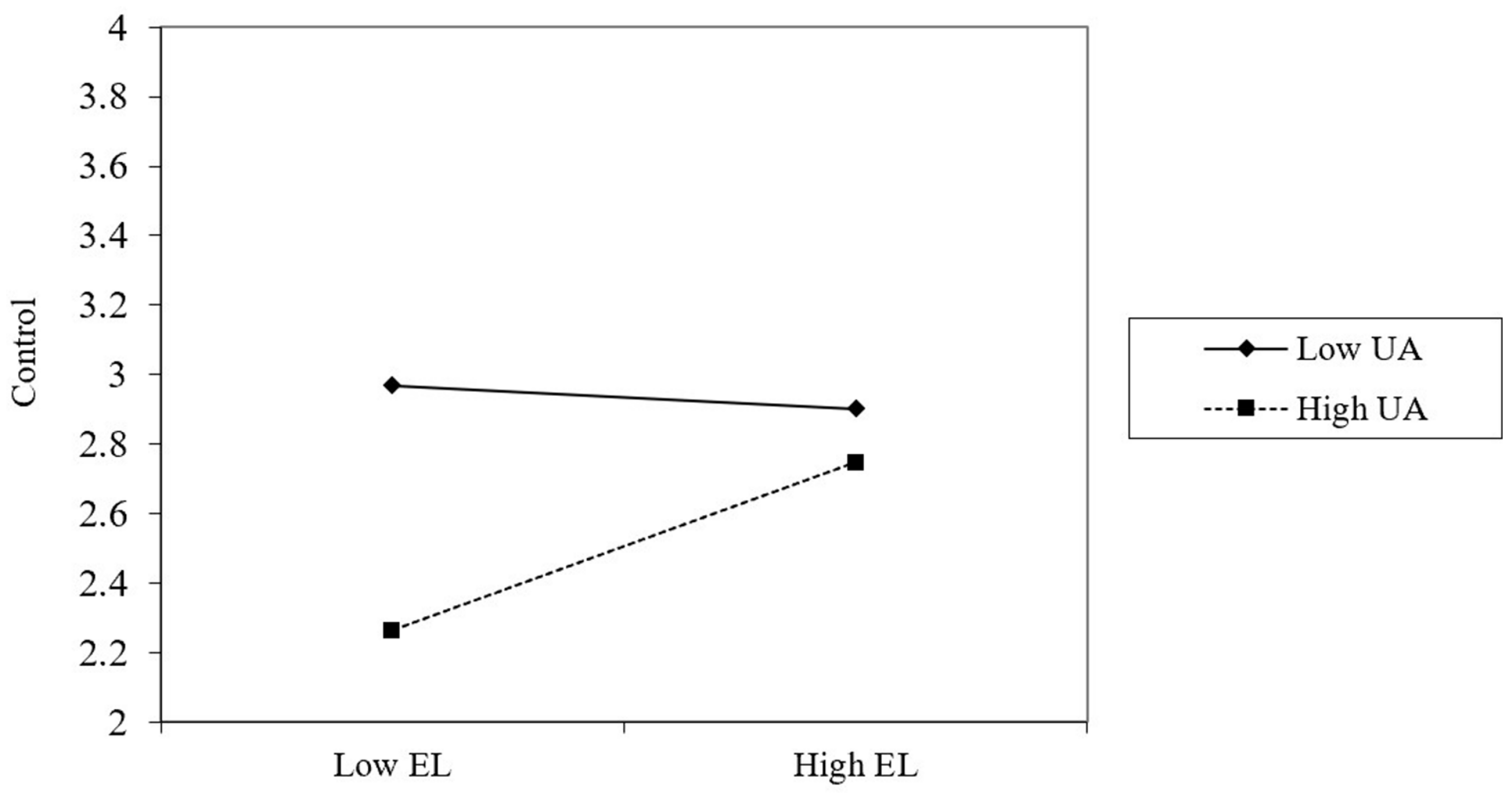
Uncertainty avoidance moderates the relationship between empathetic leadership and control.

**Figure 3:**
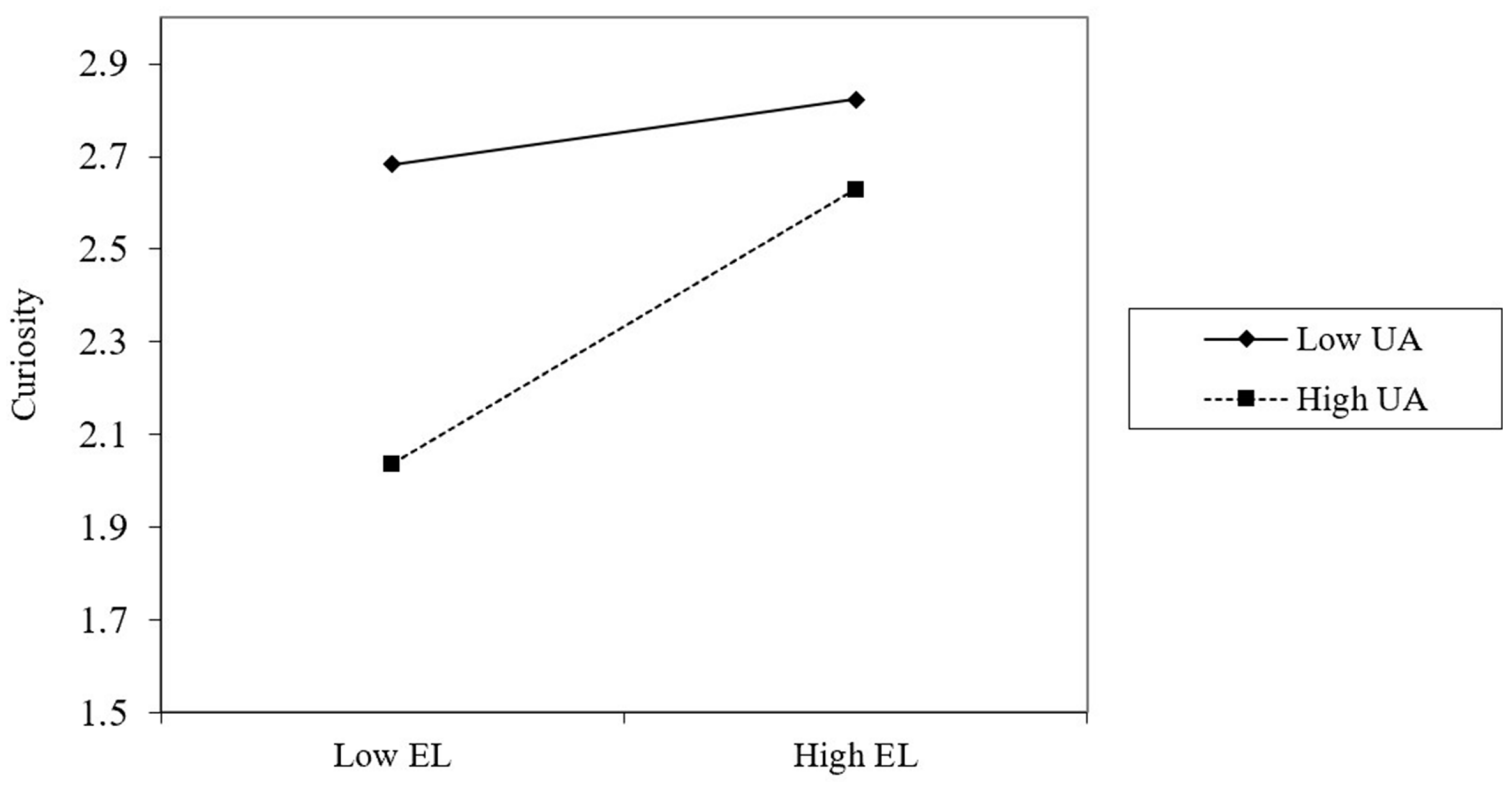
Uncertainty avoidance moderates the relationship between empathetic leadership and curiosity.

**Figure 4:**
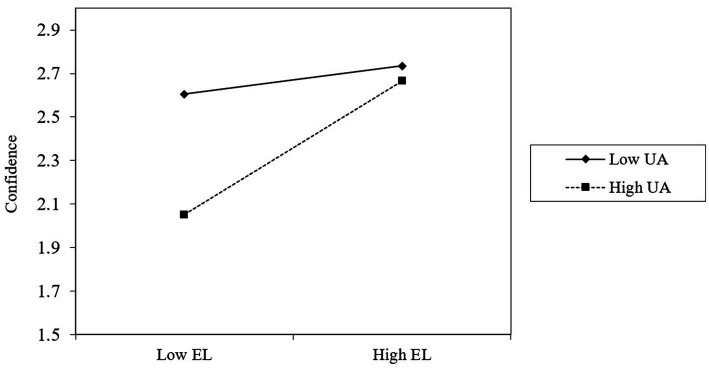
Uncertainty avoidance moderates the relationship between empathetic leadership and confidence.

To test hypothesis 3, we followed the suggestion by Cohen et al. (2003), for which the variables need to be mean-centered before simple slope calculation. We followed the PROCESS procedure outlined by Hayes (2013) to test the conditional indirect effect at different levels. As reported in Table 4 (M3, M6, M9, M12, and M15), the interaction between empathetic leadership and uncertainty avoidance was positively correlated with career adaptability (concern, control, curiosity, and confidence). This indicates that uncertainty avoidance moderated the relationship between empathetic leadership and career adaptability (*r* = 0.11, *p* < 0.05), concern (*r* = 0.11, *p* < 0.05), control (*r* = 0.11, *p* < 0.05), curiosity (*r* = 0.11, *p* < 0.05), and confidence (*r* = 0.10, *p* < 0.05). For intuitiveness, we plot the moderation effect of uncertainty avoidance. As shown in Figures 1–4, hypothesis 3a, 3b, 3c, 3d were supported.

**Figure 5:**
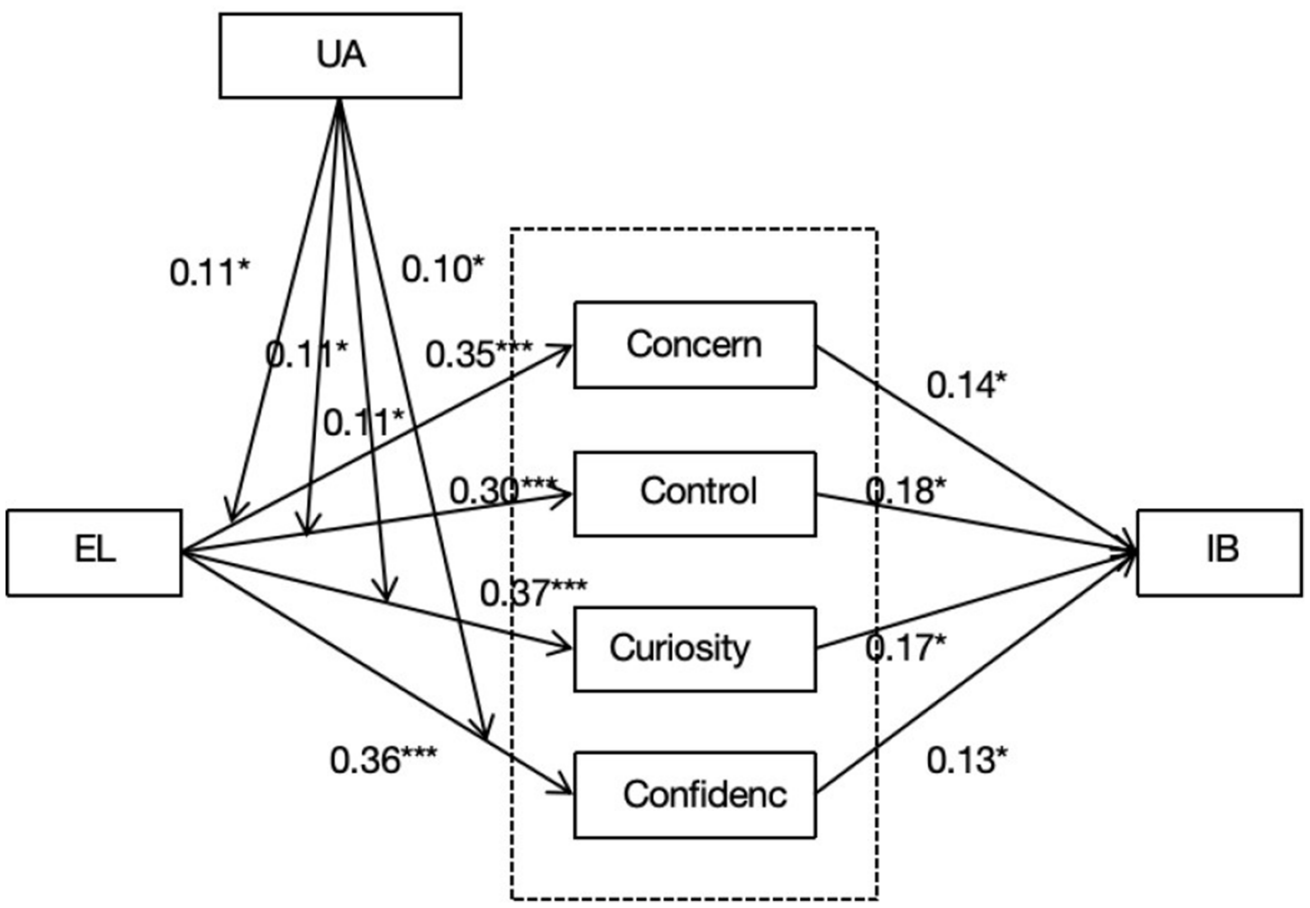
Unstandardized estimates for the hypothetical model.

Hypothesis 4 predicts that the indirect effect of empathetic leadership on innovative behavior via career adaptability (concern, concern, control, and confidence) is moderated by uncertainty avoidance. Our study adopted Mplus8.3, and the data revealed the indirect effect in moderated mediation model (Table 7). Table 7 reveals that at a high uncertainty avoidance (one standard deviation more than the mean level), the indirect effect of empathetic leadership on innovative behavior via career adaptability (*B* = 0.26, SE = 0.04, CI = [0.18, 0.34]), concern (*B* = 0.21, SE = 0.04, CI = [0.13, 0.29]), control (*B* = 0.20, SE = 0.04, CI = [0.13, 0.28]), curiosity (*B* = 0.22, SE = 0.04, CI = [0.15, 0.31]), and confidence (*B* = 0.19, SE = 0.03, CI = [0.13, 0.26]) is significant. Although this indirect effect was significant because uncertainty avoidance was low (one standard deviation less than mean level), the coefficient of indirect effect was lower. Moreover, the indirect effect of empathetic leadership on innovative behavior via career adaptability (*B* = 0.11, SE = 0.04, CI = [0.03, 0.20]), concern (*B* = 0.09, SE = 0.04, CI = [0.01, 0.17]), control (*B* = 0.07, SE = 0.04, CI = [0.01, 0.15]), curiosity (*B* = 0.10, SE = 0.04, CI = [0.03, 0.19]), and confidence (*B* = 0.09, SE = 0.03, CI = [0.03, 0.16]) is significant. Thus, hypothesis 4a, 4b, 4c, 4d were supported. We also tested index of moderated-mediation: concern (index = 0.05, SE = 0.02, CI = [0.01, 0.10]), control (index = 0.05, SE = 0.02, CI = [0.01, 0.10]), curiosity (index = 0.05, SE = 0.02, CI = [0.01, 0.09]), confidence (index = 0.04, SE = 0.02, CI = [0.001, 0.08]). And we tested the difference between the two effects (high moderator-low moderator) were significant. Figure 5 summarizes the model results of these analyses.

**Table 7:** Moderated mediating effect of uncertainty avoidance.

Variables	Level of uncertainty avoidance	Indirect effect	SE	*p*-value	95%CI
Career adaptability	High moderator	0.30	0.05	0.00	[0.21, 0.39]
	Low moderator	0.13	0.05	0.01	[0.04, 0.23]
Difference	high- low	0.17	0.07	0.01	[0.03, 0.30]
Concern	High moderator	0.25	0.05	0.00	[0.16, 0.35]
	Low moderator	0.11	0.05	0.03	[0.02, 0.21]
Difference	High- low	0.15	0.07	0.03	[0.02, 0.29]
Control	High moderator	0.24	0.05	0.00	[0.16, 0.34]
	Low moderator	0.09	0.05	0.06	[0.00, 0.18]
Difference	High- low	0.16	0.06	0.01	[0.04, 0.29]
Curiosity	High moderator	0.27	0.05	0.00	[0.18, 0.37]
	Low moderator	0.13	0.05	0.01	[0.04, 0.23]
Difference	High- low	0.14	0.07	0.03	[0.02, 0.27]
Confidence	High moderator	0.23	0.04	0.00	[0.16, 0.32]
	Low moderator	0.11	0.04	0.01	[0.04, 0.20]
Difference	High- low	0.12	0.06	0.04	[0.01, 0.24]

## 5 Discussion and conclusion

In our study, we looked at how empathetic leadership affects innovative behavior. Based on career construction theory and conservation of resources theory, we also tested the hypothesis that concern, control, curiosity, and confidence play mediating roles in linking empathetic leadership (career adaptivity) to innovative behavior (career adapting). In addition, this study found that uncertainty avoidance does not always have negative effects. People with a high uncertainty avoidance tendency may be dependent more on empathetic leadership to improve their career adaptability, resulting in their innovative behavior increasing.

### 5.1 Empathetic leadership and employees’ innovative behavior

The research results support hypothesis 1, which propounds that empathetic leadership promotes employees’ innovative behavior. The role of positive leadership in promoting innovative behavior has been discussed in the literature (Iqbal et al., 2022; AlMulhim and Mohammed, 2023). Empathetic leadership, as a new type of positive leadership, mainly involves expressing empathy to employees, provides them with ways and means to obtain resources, and then promotes their innovative behavior. The findings of this study are consistent with those of Kock et al. (2019), which confirmed that empathetic leadership can promote job innovation. However, relevant literature on empathetic leadership is scant. Specifically, the advantages of empathetic leadership have not been discussed. Our results not only corroborate the literature but also enrich the research result variables of empathetic leadership (Bani-Melhem et al., 2021). This study also enriches the impact of different types of leadership styles on employee behavior.

Moreover, career construction is regarded as a series of attempts to integrate self-concept into work roles while adapting to the repeated transitions from school to work and from job to job while meeting the challenges these pose. However, most scholars have focused on college students when discussing the future career construction of student groups (Zhang et al., 2021). However, research on the career construction for in-service staff is still lacking. This study took enterprise employees as the research object, and the results confirmed that empathetic leadership positively predicts innovative behavior. This indicates that in enterprises, leaders can also adopt empathetic behaviors to make employees perceive the support of leaders and promote positive behaviors among them.

### 5.2 Mediating role of career adaptability

The research results also support hypothesis 2, which states that the four career adaptability resources have a mediating effect between empathetic leadership and innovative behavior. Kock et al. (2019) revealed that attitude and emotion are important mediating mechanisms for empathetic leadership to influence employee behavior, but they ignored the importance of resources. The four types of adaptation resources can mediate between adaptivity and adapting (Sverko and Babarovic, 2019). Empathetic leadership can increase innovative behavior by providing support to employees and helping them obtain resources conducive to work according to career construction theory and conservation of resources theory. For example, empathetic leadership can inspire employees to explore new things and increase their confidence in solving problems. These resources will make employees more adaptable to their current jobs or tasks and improve their innovative behaviors. This study also found that the mediating effect of curiosity slightly exceeds that of the other three resources. This may be because it is important to remain curious about work and other things and to constantly explore new things when undertaking innovative activities. Our study can elucidate models and adaptability.

### 5.3 Moderating role of uncertainty avoidance

Consistent with hypotheses 3 and 4, the data results reveal that uncertainty avoidance moderates the relationship between empathetic leadership and career adaptability, as well as the impact of empathetic leadership on innovative behavior through career adaptability. Previous studies suggested that the positive relationship between positive leadership and employees’ behavior would be weakened under high uncertainty avoidance (Wang, 2020), as some scholars have suggested that employees with high uncertainty avoidance tend to abide by rules, regulations, and organizational policies while seeking help and guidance from their superiors to avoid uncertain situations (Afsar and Masood, 2018). Therefore, these individuals tend to be insensitive to authorization, preferring to rely on deterministic strategies and behaviors to achieve deterministic outcomes. In contrast, people with low levels of uncertainty avoidance are very happy to accept delegation and open organizational environments, and work more flexibly because they are more sensitive to authorization (Su et al., 2022). For example, Sheikh et al. (2013) proposed that uncertainty avoidance by subordinates moderates the relationship between transformational leadership and employee’s job involvement, making this relationship more robust for those with lower levels of uncertainty avoidance.

However, some scholars have suggested that employees tend to avoid uncertainty and ambiguity, but this does not prevent employees from preferring creative ideas (Zhang and Zhou, 2014). Uncertainty avoidance should not be confused with risk avoidance because uncertainty avoidance does not refer to an individual’s willingness to take or avoid risks. Rather, it indicates the extent to which individuals are more willing to understand the views and expectations of their supervisors (Hofstede, 2001; Taras et al., 2010). Therefore, when expectations are clear, high uncertainty avoidance individuals may have more mental resources to experience cognitive flexibility and creativity than when expectations and boundaries are not clear (Inkson et al., 1970). For example, Watts et al. (2020) proposed that compared to low uncertainty avoidance countries, high uncertainty avoidance countries will exhibit a stronger relationship between managerial transformational leadership and individual innovation.

Consistent with Watts et al. (2020)‘s findings, the results of this study confirmed that high uncertainty avoidance instead strengthened the impact of empathetic leadership on innovative behavior. This result is mainly due to the psychological factors of resource protection and fear of resource damage. Therefore, employees with high uncertainty avoidance will rely more on the support and help provided by empathetic leaders because they believe that leaders will help them reduce the loss of resources and provide them with new resources to make up for the loss of other resources. This study enriches the research on uncertainty avoidance and positive leadership (Watts et al., 2020). It also addresses Kock et al.’s (2019) recommendation to consider the moderating effect of cultural factors on empathetic leadership in the future. Meanwhile, Al-Ghazali’s (2020) research examined the career construction model but did not consider different cultural backgrounds. Our research also addresses Al-Ghazali’s (2020) recommendation to verify the applicability of the career construction model under different cultural dimensions.

## 6 Practical implications

First, organizations should cultivate a positive leadership style that is empathetic and creates a caring climate for employees. Empathy is an important element of positive leadership that needs to be learned and honed through continuous effort (Ciulla, 2010). Leaders can improve their empathy and communication skills through training. Specifically, training can revolve around good listening skills, good communication skills, and the ability to understand employee emotions (Bani-Melhem et al., 2021). Leaders can provide emotional care and support to employees by expressing genuine care to meet their expectations (Haynie et al., 2019). Moreover, leaders need to place themselves in the position of the employees, learn to be good listeners, and practice empathizing with and encouraging subordinates (Yue et al., 2023). When employees work in a caring and supportive environment, they are more motivated to create and enhance personal value, which benefits the organization. Similarly, as empathetic leadership prioritizes support for subordinates’ psychological and security needs (Kock et al., 2019), enterprises can stimulate employees with high uncertainty avoidance to improve their adaptability by investing in empathic leadership, thus achieving the maximum benefits of promoting innovative behavior.

Second, organizations can encourage employees to take responsibility for their careers by optimizing the career environment and arranging career guidance counselors for employees. As proposed in career construction theory, people develop their careers by forcing personal significance on their past and present experiences as well as future aspirations (Blokker et al., 2019). This process of self-construction requires individuals to possess sufficient skills and knowledge (Savickas, 2002). Organizations and career counselors can provide relevant skills training and interventions, including participating in planning, decision-making, exploration, and problem-solving (Savickas, 2005), to promote employee adaptability to the changing work environment and cultivate positive work behaviors. Employees should also participate in more career development training courses conducted by the organization in their daily work to establish career development plans and solve personal career development problems through the career development consulting department.

## 7 Limitation and future research directions

Although this study provides several research contributions, there are still several shortcomings that need to be solved in the future. First, although our study adopts a questionnaire design with multiple time points and multiple data sources, the design is still cross-sectional and incapable of establishing the causal relationships among variables. In the future, researchers can utilize longitudinal (Ployhart and Vandenberg, 2010) or experimental research methods to further explore the causal relationship between variables (Al-Ghazali, 2020).

Second, our study only discusses the effect of empathetic leadership on innovative behavior. As a kind of positive leadership, empathetic leadership may also produce other positive outcome variables. In the future, we can discuss the relationship between empathetic leadership, job performance and career calling.

Third, this study collected employee data from different industries and examined the mediating effect of adaptability from four aspects: attention, control, curiosity, and confidence. It was found that the mediating effect of curiosity resources was higher than that of other resources. However, this study did not compare which adaptability resources employees in different industries need more. Future research needs to examine the mediating effects of different forms of adaptability in multicultural contexts, and discuss which adaptive resources play a more important role (Karacan-Ozdemir and Ayaz, 2022). Our study only considers the intermediary mechanism between leadership and innovation from the perspective of resources (adaptability), and future studies can further discuss the relationship between the two from the perspectives of attitude and emotion.

Fourth, this study discussed empathetic leadership (career adaptivity), career adaptabilities and innovative behavior (career adapting) of the career construction model rather than the entire model. We encourage future researchers to consider adaptivity, adaptabilities, adapting, and adaptation when testing the complete model (Tokar et al., 2020). This will help to gain a deeper understanding of the career construction model.

Fifth, our study only discussed the moderating role of uncertainty avoidance from the individual perspective and found that uncertainty avoidance positively moderates the influence of empathetic leadership on career adaptability. However, uncertainty avoidance is originally a cultural variable. The moderating role of uncertainty avoidance at the group and organizational levels remains to be explored. Therefore, we can enrich and improve the theoretical model and provide effective guidance for enterprise management through cross-level research on uncertainty avoidance in the future. This study suggests that uncertainty avoidance enhances the relationship between leadership and positive behavior. There are also scholars who get inconsistent results. The moderating effect of uncertainty avoidance can be discussed from other theoretical perspectives in the future. In this study, uncertainty avoidance was studied by a horizontal data collection. In the future, uncertainty avoidance can be studied by the longitudinal data method.

## Data availability statement

The raw data supporting the conclusions of this article will be made available by the authors, without undue reservation.

## Author contributions

GM: Writing – original draft, Conceptualization, Data curation, Formal analysis, Investigation, Methodology, Software, Validation. WW: Writing – review & editing, Conceptualization. CL: Data curation, Investigation, Writing – review & editing. JJ: Data curation, Formal analysis, Investigation, Writing – review & editing. XG: Conceptualization, Data curation, Formal analysis, Investigation, Methodology, Software.
